# Ultrasound-guided quadratus lumborum block for postoperative pain control in patients undergoing unilateral inguinal hernia repair, a comparative study between two approaches

**DOI:** 10.1186/s12871-019-0862-z

**Published:** 2019-10-17

**Authors:** Abeer Ahmed, Maher Fawzy, Mohamed A. R. Nasr, Ayman M. Hussam, Eman Fouad, Hesham Aboeldahb, Dalia Saad, Safinaz Osman, Rania Samir Fahmy, Mohamed Farid, Mohsen M. Waheb

**Affiliations:** Department of Anesthesiology, Surgical ICU and pain management, Kasr Alainy Faculty of Medicine, Cairo University, 01 El Sarayah street, El Manyal, Cairo, 11559 Egypt

**Keywords:** Quadratus lumborum block, Ultrasound-guided transmuscular quadratus lumborum blockade, Ultrasound-guided lumbar plexus technique, Ultrasound guided transversus abdominus plan block

## Abstract

**Background:**

Early postoperative ambulation and reduction of hospital stay necessitate efficient postoperative analgesia. Quadrates Lumborum Block (QLB) has been described to provide adequate postoperative analgesia after abdominal surgery. This randomized comparative trial was designed to compare the duration of analgesia provided by two different QLB approaches; the posterior QLB (QLB-2) and transmuscular QLB (QLB-3) in patients undergoing surgical repair of unilateral inguinal hernia.

**Methods:**

Forty patients, aged from 18 to 50 years, ASA physical status I or II, scheduled for unilateral inguinal hernia repair were enrolled. At the end of the surgical procedure and before recovery from general anesthesia, Patients were randomly assigned into two groups to receive either posterior QLB (Group QLB-2) or transmuscular QLB (Group QLB-3) using 20 ml 0.25% bupivacaine. Duration of analgesia, postoperative VAS and postoperative opioid consumption were recorded.

**Results:**

Duration of block was significantly longer in QLB-3 group when compared to QLB-2 group (20.1 + 6.2 h versus 12.0 + 4.8 respectively) with *P* value of < 0.001. A statistically significant lower VAS score was recorded in QLB-3 group immediately and 12 h postoperative. QLB-3 group showed a statistically significant delayed time of first analgesic request and less postoperative morphine consumption with P value of < 0.001 and 0.001 respectively.

**Conclusions:**

Ultrasound guided postsurgical transmuscular approach of QLB (QLB-3) using 20 ml 0.25% bupivacaine produces more postoperative analgesic effect and less postoperative opioid consumption when compared to posterior QLB approach (QLB-2) in patients underwent unilateral inguinal hernia repair under general anesthesia.

**Trial registration:**

ClinicalTrials.gov identifier: NCT03526731- on 16 May 2018.

## Background

The ultrasound-guided (USG) quadratus lumborum block QLB was first described by Rafael Blanco in a presentation at ESRA 2007 at the XXVI Annual ESRA Congress in Valencia, Spain. Blanco described a potential space posterior to the abdominal wall muscles and lateral to the quadratus lumborum muscle (QL) where Local anesthetics (LA) can be injected [1]. This technique provides analgesia after abdominal surgeries due to spread of LA from its lumbar deposition cranially into the thoracic paravertebral space (TPVS) where lateral and anterior cutaneous branches from Th7 to L1 nerves can be blocked [2, 3]. This was proved later by Carney et al. [3] who found traces of contrast agent in the TPVS following the block.

Several approaches have been described for QLB. Lateral QLB (or QLB-1) where local anesthetic is injected at the anterolateral border of the QL muscle. Posterior QLB (or QLB-2) where LA is injected at the junction of QL muscle with the transversalis fascia [4]., Another novel approach is the transmuscular QLB (or QLB-3), where the needle is advanced through the QL muscle, penetrating the ventral proper fascia of the QL muscle and LA is finally injected between the QL muscle and Psoas Major (PM) muscle [5, 6]. It is thought that this approach (QLB-3) does not result in redundant antero-lateral spread of the LA [7].

To the best of our knowledge, there is neither agreement about the best approach for QLB block nor their analgesic efficacy have been compared. This randomized comparative trial was designed to compare the duration of analgesia provided by the posterior QLB (QLB-2) versus transmuscular QLB (QLB-3) in patients undergoing surgical repair of unilateral inguinal hernia. We hypothesized that transmuscular QLB (QLB-3) could provide a longer duration of analgesia when compared to posterior QLB (QLB-2).

## Methods

This randomized comparative study was conducted in the general surgery operating unit of Cairo University Hospitals after obtaining an approval of the Research Ethics Committee of the Faculty of Medicine, Cairo University (email: kasralainirec@gmail.com ID: N-13-2016) and registration on ClinicalTrials.gov identifier: NCT03526731- on 16 May 2018. The Consolidated Standards of Reporting Trials (CONSORT) Guidelines were followed. A written informed consent was obtained from all patients. Forty patients, aged from 18 to 50 years, ASA physical status I or II, who were scheduled for unilateral inguinal hernia repair under general anesthesia were enrolled. Patients with systemic hypertension, cardiovascular disease, cerebrovascular insufficiency, coagulation abnormities, renal or hepatic insufficiency, infection at the injection site, strangulated hernia and hypersensitivity to the local anesthetics were excluded from the study. Once enrolled; patients were randomly assigned into two equal groups: QLB-2 group (received posterior QLB) and QLB-3 group (received transmuscular QLB). Randomization was performed using an online random number generator. Concealment was achieved using sealed opaque envelopes.

On arrival to the operating room, an intravenous line was inserted, 1–2 mg midazolam was given and 500 ml Ringer acetate infusion was started. A five-lead electrocardiogram, a pulse oximeter and a noninvasive blood pressure monitor were applied. General anesthesia was induced using fentanyl 2 μg/kg, propofol 2 mg/kg, and atracurium 0.5 mg/kg to facilitate endoracheal intubation. Anesthesia was maintained using isoflurane with E_T_ concentration of 1–1.5% and atracurium besylate top-up doses 0.1 mg/kg were given based on the response to train-of-four ulnar nerve stimulation. Mechanical ventilation was adjusted to keep the E_T_CO_2_ at 30–35 mmHg. All patients received one gram of paracetamol as intravenous infusion with the start of skin closure.

At the end of the surgical procedure and before recovery from general anesthesia, all patients were positioned in lateral position with the side to be anesthetized faced upwards, sterilized and covered with sterile sheets. Aseptic precautions were taken by wearing sterile gowns and gloves. Ultrasound (ACUSON Freestyle, Siemens Medical Solutions, Inc. USA.) was used; with broadband (5–8 MHz) convex probe covered with sterile plastic sheath. The probe was placed in the mid axillary line cranially to the iliac crest to identify the three muscles of the anterior abdominal wall (transversus abdominis, internal oblique, and external oblique), then scan dorsally keeping the transverse orientation until observing that the transversus abdominus muscle becomes aponeurotic, and this aponeurosis was followed until the QL muscle was clearly visualized with its attachment to the lateral edge of the transverse process of L4 vertebral body and visualize the thoracolumbar fascia at the lateral edge of the QL muscle.

### For QLB-2 group (posterior QLB)

**Fig. 1:**
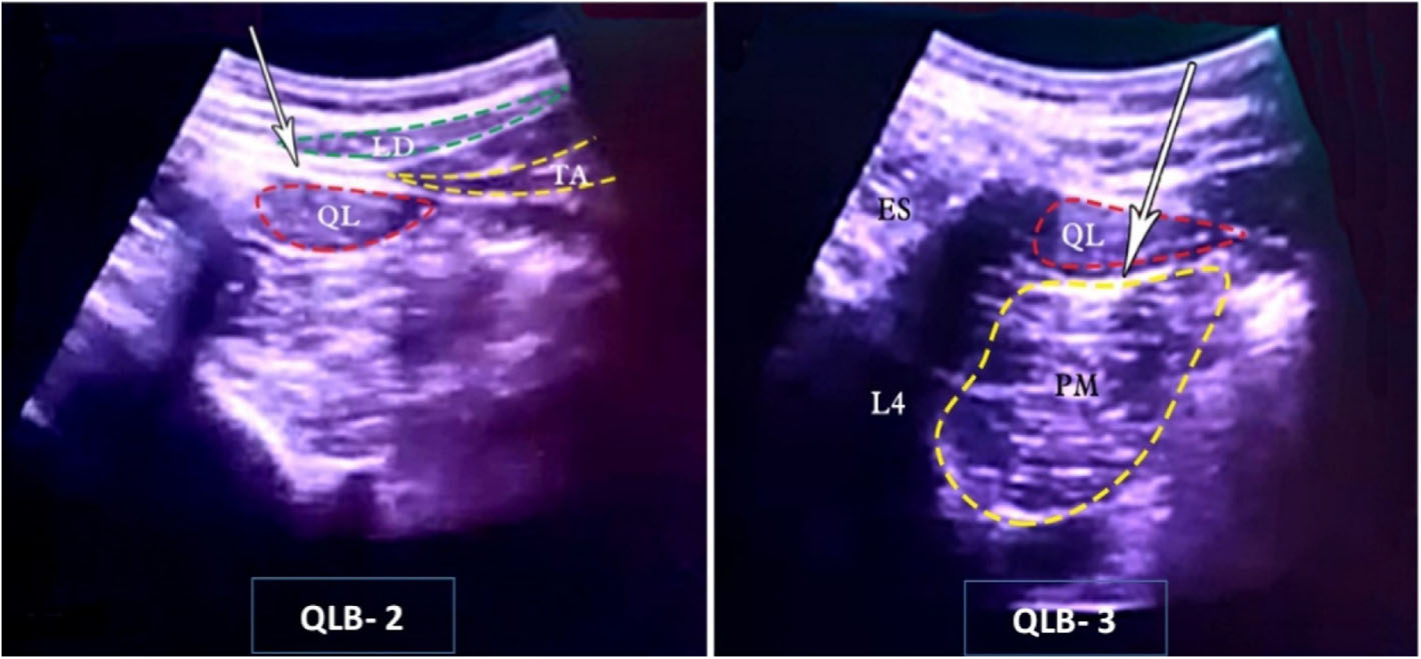
U/S image for QLB 2&3

The needle (20G spinal needle filled with glucose 5% with bevel up facing the ultrasound probe) was inserted in-plane from anterior to posterior and the tip of the needle was advanced towards the posterior border of the QL muscle, between the QL and the latissimus dorsi (LD) muscles, 1 ml test dose of saline was injected to confirm correct needle-tip position, and then this was followed by injection of 20 ml of 0.25% bupivacaine (Marcaine,Astra Zeneca, UK). (Fig. 1).

### For QLB-3 group (transmuscular QLB)

The needle (20G spinal needle filled with glucose 5% with bevel up facing the ultrasound probe) was inserted in-plane from anterior to posterior and the tip of the needle was advanced towards then through the QL muscle, penetrating the ventral proper fascia of the QL muscle. The target site for injection was the plane between QL muscle and PM muscle, 1 ml test dose of saline was injected to confirm correct needle-tip position, and then this was followed by injection of 20 ml of 0.25% bupivacaine (Marcaine, Astra Zeneca, UK). (Fig. 1).

All patients were then turned supine. Isoflurane was discontinued and the residual of the muscle relaxant was antagonized with neostigmine 0.05 μg/kg and atropine 0.02 mg/kg. The trachea was extubated once the patients showed eye opening and purposeful movement then patients were transferred to the post anesthesia care unit (PACU). All outcome measures were collected by an anesthesiologist who was not involved in block performance.

### Primary outcome

- **The duration of block (The time to first analgesic request)** which is defined as the time interval between end of LA injection and patient pain complaint (VAS > 3).

**Secondary outcomes:**

- **The duration of technique** which is defined as time interval between placements of the ultrasound probe on patient’s skin till removal of the needle after termination of the LA injection.
- **The sensory bock** was assessed using ice packs at sensory points (ipsilateral sensory assessment from T6 to L1) immediately after block and every 5 min for 30 min using 4 points scale [8] as follow: 3 if normal sensation, 2 if decreased cold sensation, 1 if absent cold/ present touch sensation and 0 if absent cold/absent touch sensation. Site of block was compared to the unblocked site. In our study, a successful block was defined as a sensory block score 0–1, and so **success rate** could be calculated.
- **Visual Analogue Score (VAS)** was recorded postoperatively at the following time intervals; immediately, 2, 6, 12, 24 h postoperative. If VAS score is more than 3, patient received intravenous morphine of 1 mg that was repeated after 20 min till VAS score reached < 3.
- **Total Morphine consumption over the first postoperative 24 h**.

Both data collector and participants were blinded to the approach used.

### Sample size calculation and statistical analysis

Our primary outcome was the duration of block that was defined as the time interval between end of LA injection and patient pain complaint (VAS > 3). We had a pilot study included 6 patients received posteriors QLB (QLB-2), the duration of block was 11 h with SD of 1.1 h. We took an assumption for clinical significance if the duration of block increased by 40%, with a study power of 80% and alpha error of 0.05, a minimum number of 18 patients was required for each group, this number was increased by 10% (to be 20 patients per group) to compensate for possible drop-outs. The G power 3.1.9.2 program was used for sample size calculation. The Statistical Package of Social Science software program (SPSS), version 21 (Chicago, IL, USA) was used for all statistical comparisons. Continuous quantitative normally distributed data was expressed as means and standard deviations (SD). Qualitative nominal data was expressed by percentage, two-way repeated measurement analysis of variance (ANOVA) was used for comparing the change of duration of analgesia between the two approaches. The ANOVA analysis was followed by Tukey post hoc tests. A *P* value of < 0.05 was considered statistically significant.

## Results

**Fig. 2:**
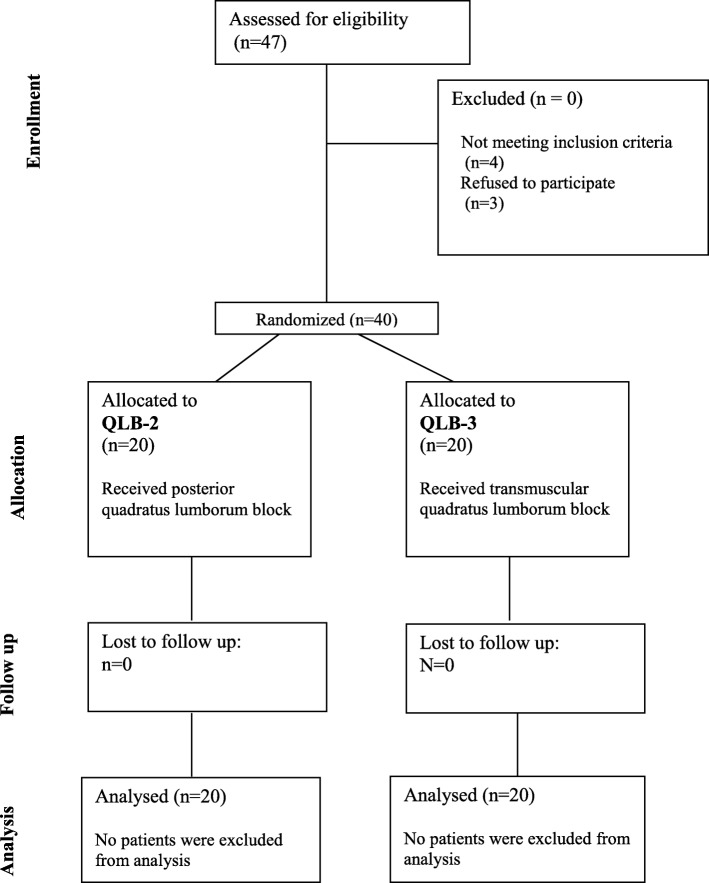
Participants flow diagram

Forty Patients, aged from 18 to 50 years old, with ASA I – II, underwent unilateral inguinal hernia repair surgery were included and analysed in the study (Fig. 2). Patients were divided into two equal groups: QLB-2 group and QLB-3 group. Patients and surgery characteristics are shown in (Table 1).

**Table 1:** Patients and surgery characteristics

Variable	QLB −2 Group *N* = 20	QLB – 3 Group *N* = 20	*P* values
Age (years)	31.9 + 7.2	29.9 + 7.0	0.364
Gender (male/female)	18/2	19/1	0.548
BMI	30.5 + 2.0	29.9 + 1.3	0.413
ASA (I/II)	6/14	4/16	0.716
Duration of surgery (min)	68.0 + 7.3	68.5 + 7.8	0.910
Side of surgery (Left/right)	10/10	11/9	0.752

Data are expressed as mean + SD or absolute numbers.

The QLB-2 group and the QLB-3 group showed no statistical significant differences in either duration of technique or the success rate, but the duration of block was significantly longer in patient received transmuscular QLB (QLB-3 group) when compared to QLB-2 group (20.1 + 6.2 h versus 12.0 + 4.8 respectively) with *P* value of < 0.001 (Table 2).

**Table 2:** Block characteristics

Variable	QLB – 2 Group *N* = 20	QLB – 3 Group *N* = 20	*P* values
Duration of technique (min)	9.8 + 1.5	10.0 + 1.4	0.646
Duration of block (time of first analgesic request. (hr)	12.0 + 4.8^a^	20.1 + 6.2^a^	< 0.001
Block success rate. N (%)	20 (100%)	20 (100%)	
Total opioid consumption over 24 h (mg)	1.9 + 0.6^a^	1.1 + 0.9^a^	0.001

Data are expressed as mean + SD or percentage. ^a^means statistically significant

The Visual Analogue Score (VAS) was compared between both groups over the first 24 h postoperatively, the comparison revealed a statistically significant lower VAS score in QLB-3 group immediately and 12 h postoperative (Table 3).

**Table 3:** VAS in both groups

Variable	QLB – 2 Group *N* = 20	QLB – 3 Group *N* = 20	*P* values
VAS immediate PO			
Range	0.0–2.0^a^	0.0–2.0^a^	0.027
Median	2.0	0.0	
VAS 2 h			
Range	0.0–2.0	0.0–2.0	0.262
Median	2.0	2.0	
VAS 6 h			
Range	2.0–4.0	0.0–4.0	0.060
Median	2.0	2.0	
VAS 12 h			
Range	2.0–6.0^a^	2.0–4.0^a^	< 0.001
Median	4.0	2.0	
VAS 24 h			
Range	2.0–6.0	0.0–4.0	0.086
Median	4.0	4.0	

Data are expressed as range & median. ^a^means statistically significant

Analgesic characteristics in form of time to first analgesic request and total morphine consumption over 24 h postoperatively were compared in both groups. The patients in QLB-3 group showed a significant delayed time to first analgesic request and less morphine consumption with P value of < 0.001 and 0.001 respectively (Table 2). In both groups, no harms in form of hematoma formation or visceral injuries were recorded.

## Discussion

The main finding of this study is that postsurgical in-plane ultrasound guided transmuscular approach of QLB (QLB-3) using 20 ml of 0.25% bupivacaine produces longer postoperative duration of analgesia and less postoperative opioid consumption when compared to the posterior approach of QLB (QLB-2) in patients undergoing unilateral inguinal hernia repair under general anesthesia.

Ultrasound guided QLB block was first described by Rafael Blanco [1]. Blanco clarified that QLB is differ from the known TAP block (Transversus Abdominal Plane block) as the latter is superficial to the transversus abdominis muscle and its aponeurosis, while the QLB is actually deep to the transversus abdominis aponeurosis [9]. In the QLB, LA spreads from its lumbar deposition cranially into TPVS, this could explain why QLB would seem to be able to alleviate both somatic and visceral pain [7, 10], and why QLB could provide analgesia after abdominal surgeries [2, 3]. On contrary, TAP block comprises infiltration into the anterior abdominal wall and hence block somatic fibers only [4]. Since Ultrasound guided QLB block was first described, several case reports in both adult [11, 12] and pediatric patients [13, 14] have been published. All proved the block efficacy in reducing the severity of postoperative pain scores and opioid consumption.

In the first randomized controlled trial designed to evaluate the analgesic efficacy of the QLB after Caesarean section [4], authors described their use of MRI with contrast to study the spread of LA from two injection points. The first point was at the anterolateral border of the QL muscle (QLB-1), while the second was at its posterior border (QLB-2). The study revealed that injection at posterior border of QL muscle (OLB-2) was associated with more predictable spread of the LA. Authors denoted that QLB-2 provided a higher safety level with less complications due to better image resolution, more superficial approach and longer distance from the intra-abdominal viscera. Authors described QLB-2 as the optimal point of injection. This could explain why the posterior QLB (QLB-2) was selected in our study.

The novel ultrasound guided transmuscular QLB (QLB-3) was described by Börglum et al., where LA was injected between the PM and QL muscles [5]. The injected LA potentially spreads cranially to reach the TPVS. The MRI performed one hour after injection revealed a clear cranial spread along the PM and QL muscles till reach beyond the arcuate ligament with less redundant antero-lateral spread [5]. The potential cranial spread of LA after transmuscular approach could be attributed to the same embryonic origin and insertion of both PM and QL muscles within the thoracic cage [15, 16]. These findings were later supported by two cadaveric studies assessed the spread of LA after 4 cases used QLB-3. These studies revealed the spread of the LA consistently to L1 and L3 nerve roots within PM and QL muscles [17, 18]. This spread is predominantly via a pathway posterior to the arcuate ligaments and into the TPVS. LA reaches the somatic nerves and the thoracic sympathetic trunk in the intercostal and paravertebral spaces. The lumbar plexus and lumbar sympathetic trunk are not affected [14, 17, 18]. This could explain the extensive thoracolumbar anesthesia following the transmuscular QLB approach.

No much clinical trials studied the transmuscular QLB (QLB-3). In two cases series involved 2 and 5 children underwent hip surgeries and pyeloplasty respectively [6, 7], it was revealed that QLB-3 could provide an effective postoperative analgesia with reduced postoperative analgesic requirement. The duration of the analgesia could last up to 24 h postoperatively [6].

In all published literatures, there is no agreement about the appropriate LA volume or concentration that can be injected for the QLB either in adults or in pediatrics [7, 11–14, 19]. Carney et al. used 0.3 to 0.6 mL/kg of LA with contrast in their anatomic study in adults to be able to detect the contrast agent in the TPVS [3]. In our study, we used 20 ml of 0.25% bupivacaine considering previous reports [17, 19], denoted that at least 20 mL of the LA at one site may be required. The safety of this volume has been confirmed by Murouchi et al. [19] who measured the LA concentration after QLB using 20 mL per side from 0.375% ropivacaine and revealed that the plasma concentration of the LA was below the toxic threshold.

In all previously mentioned studies [4, 6, 7, 11–14, 19], QLB has been used as an adjuvant either to reduce the intraoperative requirements of general anesthesia or as a part of postoperative multimodal analgesia. In our study, postsurgical QLB was performed as a postoperative pain relief maneuver. QLB has not previously used as a sole anesthetic technique and it is yet not known if QLB could completely relief both somatic and visceral pain?

To the best of our knowledge, the current study is the first RCT designed to compare the analgesic efficacy of transmuscular QLB (QLB-3) with posterior QLB (QLB-2). The results of our study showed a significant increase in the duration of postoperative analgesia with significant reduction of the total postoperative opioid consumption in patients received QLB-3 compared to those received QLB-2. Our results are in agreement with the previous explanation denoting that the transmuscular QLB has more cranial spread of the LA along the QL muscle and PM muscle without any redundant antero-lateral spread that provides more extensive thoracolumbar anesthesia [5–7].

This study may show some limitations. We did not measure the LA concentration in serum after using 20 ml of 0.25% bupivacaine. We did not use imaging studies with contrast enhancement to follow the pattern of spread of this volume of LA in both approaches. This is a unilateral surgical procedure with limited surgical incision, so both approaches are in need to be compared when the procedure is bilateral and/or with an extended surgical incision.

## Conclusions

Ultrasound guided postsurgical transmuscular approach of QLB (QLB-3) using 20 ml of 0.25% bupivacaine produces more postoperative analgesic effect and less postoperative opioid consumption when compared to posterior QLB approach (QLB-2) in patients underwent unilateral inguinal hernia repair under general anesthesia.

## Data Availability

The data that support the findings of this study are available from Cairo university hospitals; however, they are not publicly available. Data are however available from the authors upon reasonable request after permission of Cairo university hospitals.

## Abbreviations

ANOVA: Analysis of variance
ES muscle: Erector Spinae muscle
E_T_: End Tidal
E_T_CO_2_: End Tidal carbon dioxide
LA: Local anesthetic
LD muscle: Latissimus dorsi muscles
PACU: Post anesthesia care unit
PM muscle: Psoas major muscle
QL: Quadrates lumborum
QLB: Quadrates lumborum block
TAP block: Transversus Abdominal Plane block
TPVS: Thoracic paravertebral space
USG: ultrasound-guided
VAS: Visual analogue score
